# Integrated bioinformatics analysis and clinical validation identifies TNFSF14 and CD40 as novel biomarkers for chronic kidney disease progression and tubulointerstitial injury

**DOI:** 10.1007/s11255-026-05138-9

**Published:** 2026-04-25

**Authors:** Xiameng Gu, Yuqing Lu, Haonan Sha, Hanlu Zhang, Hongxin Chen, Mengyue Qiu, Xiaolan Chen

**Affiliations:** 1 Department of Nephrology, Affiliated Hospital of Nantong University, Jiangsu, 226001 China; 2 Department of Pediatric Surgery, University Medical Center Mannheim, University of Heidelberg, Mannheim, Germany

**Keywords:** Chronic kidney disease, Bioinformatics, TNFSF14, CD40, Biomarker, Renal fibrosis, Tubulointerstitial injury, Urinary biomarkers

## Abstract

**Background:**

Chronic kidney disease (CKD) is a global public health burden characterized by irreversible renal function loss and progressive fibrosis. Non-invasive biomarkers reflecting intra-renal inflammation and early tubulointerstitial injury remain an unmet clinical need. We combined bioinformatics analysis with clinical validation to characterize two immune-related genes, TNFSF14 and CD40, in CKD progression.

**Methods:**

Two independent CKD transcriptomic datasets (GSE66494, *n* = 61; GSE97709, *n* = 48) were retrieved from the GEO database. Differentially Expressed Genes (DEGs) were screened with FDR adjustment. Clinical validation was performed in 140 CKD patients (KDIGO Stages I–V) and 60 healthy controls. TNFSF14 and CD40 levels were quantified in serum/urine via ELISA, with intra-renal expression assessed via immunofluorescence in 80 biopsy specimens. Multivariable regression was used to evaluate independent predictive value.

**Results:**

TNFSF14 and CD40 were identified as core hub genes enriched in the TNF signaling pathway. Both markers were significantly elevated in serum/urine of CKD patients (Adjusted *P* < 0.05), with upregulation localized to renal tubular epithelial cells. Urinary levels increased in a CKD stage-dependent manner, positively correlating with serum creatinine/BUN and inversely with eGFR. Urinary TNFSF14 and CD40 were independent predictors of advanced CKD, with a combined diagnostic model achieving an AUC of 0.892 (95% CI: 0.851–0.933).

**Conclusions:**

TNFSF14 and CD40 are robust molecular signatures of tubulointerstitial injury, and their urinary levels serve as non-invasive biomarkers for CKD detection and risk stratification.

**Supplementary Information:**

The online version contains supplementary material available at https://doi.org/10.1007/s11255-026-05138-9.

## Introduction

Chronic kidney disease (CKD) has emerged as a formidable public health challenge worldwide, with its prevalence rising rapidly alongside global population aging and the growing epidemic of metabolic disorders including diabetes and hypertension. Recent epidemiological data from the Global Burden of Disease study estimate that nearly 10% of the global population lives with CKD, which has become one of the leading causes of death among non-communicable diseases globally [1, 2]. Though the initiating etiologies of CKD are highly heterogeneous, ranging from metabolic and hypertensive injury to autoimmune and genetic disorders, the downstream pathological progression of the disease converges on a common pathway: persistent chronic inflammation, peritubular capillary rarefaction, and progressive tubulointerstitial fibrosis [3].

Serum creatinine and estimated glomerular filtration rate (eGFR) remain the cornerstone metrics for CKD diagnosis and staging in current clinical practice. In recent decades, several novel kidney injury biomarkers have been identified, most notably Neutrophil Gelatinase-Associated Lipocalin (NGAL) and Kidney Injury Molecule-1 (KIM-1). While these markers show high accuracy for detecting acute tubular necrosis in the setting of acute kidney injury, their performance in monitoring chronic, slowly progressive renal fibrosis remains inconsistent and suboptimal [4]. More importantly, these markers largely serve as generic indicators of renal structural damage, rather than providing insights into the specific immune-mediated pathogenic pathways driving disease progression. There is a critical lack of mechanism-based biomarkers for CKD: molecules that can not only detect the presence of renal injury, but also reveal the underlying inflammatory pathways driving fibrogenesis. The identification of such pathway-specific biomarkers would not only improve risk stratification of CKD patients, but also help identify subsets of patients who may benefit from targeted immunotherapies [5, 6].

Current CKD staging relies almost exclusively on eGFR and albuminuria, as recommended by international clinical practice guidelines [7, 8]. However, these well-established metrics have well-recognized diagnostic limitations: they are essentially lagging indicators of kidney disease, which cannot capture early cellular stress or active inflammatory processes within the renal parenchyma before significant functional decline occurs [9]. By the time a reduction in eGFR or elevation in albuminuria is detected, substantial irreversible structural damage to the kidney has often already developed. This critical clinical gap highlights the urgent need for non-invasive “liquid biopsy” biomarkers: molecules that can be readily detected in urine or blood, and accurately reflect real-time molecular and pathological changes in the tubulointerstitium before the onset of irreversible fibrosis [10, 11].

At the mechanistic level, the tumor necrosis factor (TNF) superfamily lies at the critical intersection of innate immunity, inflammatory response, and tissue remodeling [12]. Within this superfamily, TNFSF14 (also known as LIGHT) and CD40 have attracted increasing attention not only as key immune co-stimulatory molecules, but also as potential drivers of renal pathological damage. Emerging preclinical evidence has shown that TNFSF14 acts as a key checkpoint in vascular injury and metabolic disorders [13], while the CD40-CD40L signaling axis plays a well-documented role in promoting macrophage activation and tubular epithelial–mesenchymal transition (EMT) in the kidney [14, 15]. Despite these mechanistic insights from preclinical models, the clinical utility of TNFSF14 and CD40 as non-invasive biomarkers for CKD remains largely uncharacterized in well-powered clinical cohorts.

To address this critical gap between transcriptomic discoveries and clinical translation, we performed a multi-step translational study in the present work. We integrated comprehensive bioinformatics analysis of two independent GEO datasets with rigorous clinical validation in a single-center cohort, to systematically characterize the expression patterns of TNFSF14 and CD40 in CKD. The primary aim of our study was to determine whether these two immune-related molecules are upregulated in the renal tubulointerstitium of CKD patients, and whether their urinary concentrations can serve as robust, non-invasive biomarkers for CKD progression.

## Materials and methods

### Data mining and differential expression analysis

Gene expression profiles were retrieved from the NCBI Gene Expression Omnibus (GEO) database. Datasets GSE66494 and GSE97709 were selected to compare CKD tissues against normal controls. GSE66494 contains microarray data (Agilent platform GPL6480) from 53 renal biopsy specimens and 8 controls, capturing gene expression changes linked to interstitial fibrosis. GSE97709 employs next-generation RNA sequencing to profile circulating RNA in 48 subjects (28 ESRD, 8 CKD, 12 healthy controls).

Raw data were processed for stringent quality control. Quantile normalization and log2 transformation were applied to align the distributions of probe intensities and read counts. To address the significant technical variance and batch effects arising from the different experimental platforms (microarray vs. RNA-seq), the ComBat algorithm from the sva R package was utilized to harmonize the expression matrices. The limma package (version 3.40.6) was used to screen for Differentially Expressed Genes (DEGs). Significance was defined as an absolute log2 Fold Change (FC) ≥ 1.5. To strictly control for multiple testing, P-values were adjusted using the Benjamini–Hochberg false discovery rate (FDR), with an FDR < 0.05 deemed statistically significant. Overlaps between datasets were visualized using Venny 2.1. Functional annotation was performed via Gene Ontology (GO) and Kyoto Encyclopedia of Genes and Genomes (KEGG) enrichment analyses utilizing the clusterProfiler package. Protein–protein interaction (PPI) networks were mapped using the STRING database.

### Clinical cohort description and ethical approval

This study was conducted in strict accordance with the Declaration of Helsinki and received formal approval from the Ethics Committee of the Affiliated Hospital of Nantong University (Approval No. 2023-Y114-01). Written informed consent was obtained from all participants prior to enrollment. A total of 140 patients admitted to the Department of Nephrology with a confirmed diagnosis of CKD were enrolled. Based on KDIGO guidelines, patients were stratified by eGFR into Stages I–V: Stage I (*n* = 30), Stage II (*n* = 31), Stage III (*n* = 28), Stage IV (*n* = 25), and Stage V (*n* = 26). Within this cohort, 80 patients underwent diagnostic renal biopsy, representing four primary etiologies: diabetic nephropathy, IgA nephropathy, membranous nephropathy, and FSGS (*n* = 20 each). Exclusion criteria encompassed acute kidney injury (AKI), active systemic infection, active malignancy, or incomplete clinical records. A control group comprising 60 healthy individuals with confirmed normal renal function was recruited concurrently from the physical examination center.

### Serum and urine sample processing and immunoassays

Peripheral blood (5 mL) and mid-stream urine (50 mL) were collected from all participants. Following centrifugation at 3000 × g for 15 min at 4 °C to eliminate cellular debris, supernatants were aliquoted into RNase/DNase-free tubes and stored at −80 °C until quantification.

Target protein concentrations were quantified using commercially available, highly sensitive enzyme-linked immunosorbent assay (ELISA) kits to ensure the detection of trace biomarker shedding. Human TNFSF14 was measured using the TNFSF14 ELISA kit (MultiSciences, Cat# EK1217-01, Hangzhou, Zhejiang, China), which possesses a detection range of 31.25–2000 pg/mL and an analytical sensitivity of < 1.87 pg/mL. Human CD40 was measured using the sCD40L ELISA Kit (MultiSciences, Cat# EK188-01, Hangzhou, Zhejiang, China) featuring a detection range of 62.5–4000 pg/mL and a sensitivity of < 4.09 pg/mL. All assays were executed in duplicate according to the manufacturer’s precise protocols, and optical densities were measured at 450 nm using a standard microplate reader.

### Histopathology and immunofluorescence

Renal biopsy tissues from the 80 biopsied CKD patients were utilized. Control renal tissues (*n* = 20) were harvested from the unaffected, para-cancerous margins of patients undergoing nephrectomy for localized renal carcinoma. Tissues were paraffin-embedded and sectioned at 4 μm thickness. For immunofluorescence, sections underwent standard de-paraffinization, rehydration, and heat-induced antigen retrieval.

Following a blocking step with 5% BSA, sections were incubated overnight at 4 °C with primary antibodies. To ensure reproducibility, highly validated antibodies were selected: rabbit polyclonal anti-TNFSF14 (Cat# BS-2462R, Thermo Fisher Scientific; diluted 1:200) and rat monoclonal anti-CD40, clone 1C10 (Cat# 14-0401-82, Thermo Fisher Scientific; diluted 1:100). Target specificity for these antibodies in immunohistochemical applications has been stringently verified by the manufacturer. This was followed by incubation with appropriate fluorophore-conjugated secondary antibodies. Nuclei were counterstained with DAPI, and fluorescence was visualized using a NIKON ECLIPSE C1 microscope. ImageJ software was employed for quantitative analysis, performing background subtraction and calculating the mean fluorescence intensity per field.

### Statistical analysis

Comprehensive data analysis was conducted using SPSS 25.0 (IBM Corp, Armonk, NY, USA) and R software. The assumption of normality for all continuous variables was formally assessed using the Shapiro–Wilk test. Non-normally distributed continuous variables were log-transformed prior to parametric testing. Missing data, which accounted for < 5% of the total dataset, were handled using multiple imputation to preserve statistical power. Normally distributed data are presented as mean ± standard deviation (SD). For baseline comparisons between two groups, the Student’s *t*-test was utilized; for multi-group comparisons, a one-way analysis of covariance (ANCOVA) was applied to adjust for baseline imbalances. Non-normally distributed data were evaluated using the non-parametric Mann–Whitney *U* or Kruskal–Wallis tests.

To rigorously evaluate the relationship between gene markers and clinical parameters, we adhered to stringent statistical reporting guidelines. To avoid the ambiguity of vaguely “adjusting for confounders,” Partial Correlation Analysis and multivariable logistic regression models were performed with explicitly defined covariates. The multivariable models were adjusted for age (continuous), gender (categorical), body mass index (continuous), diabetes mellitus (categorical), and hypertension (categorical). The adjusted odds ratios (OR) derived from the logistic regression were visualized using a forest plot. The goodness-of-fit of the logistic regression model was assessed using the Hosmer–Lemeshow test and McFadden’s pseudo R-squared.

To control the family-wise error rate associated with multiple testing, the Benjamini–Hochberg false discovery rate (FDR) adjustment was strictly applied during the transcriptomic DEG screening. Furthermore, *P*-values derived from all clinical correlation matrix analyses were adjusted using the Bonferroni correction. Receiver operating characteristic (ROC) curves with 95% confidence intervals (CIs) were generated to evaluate diagnostic performance. A post hoc power calculation confirmed that the total sample size of 200 provided > 80% statistical power to detect significant differences in biomarker expression between the primary groups at a two-sided alpha level of 0.05. Statistical significance was definitively established as an adjusted *P*-value < 0.05.

## Results

### Transcriptomic profiling uncovers a robust CKD signature

**Fig. 1:**
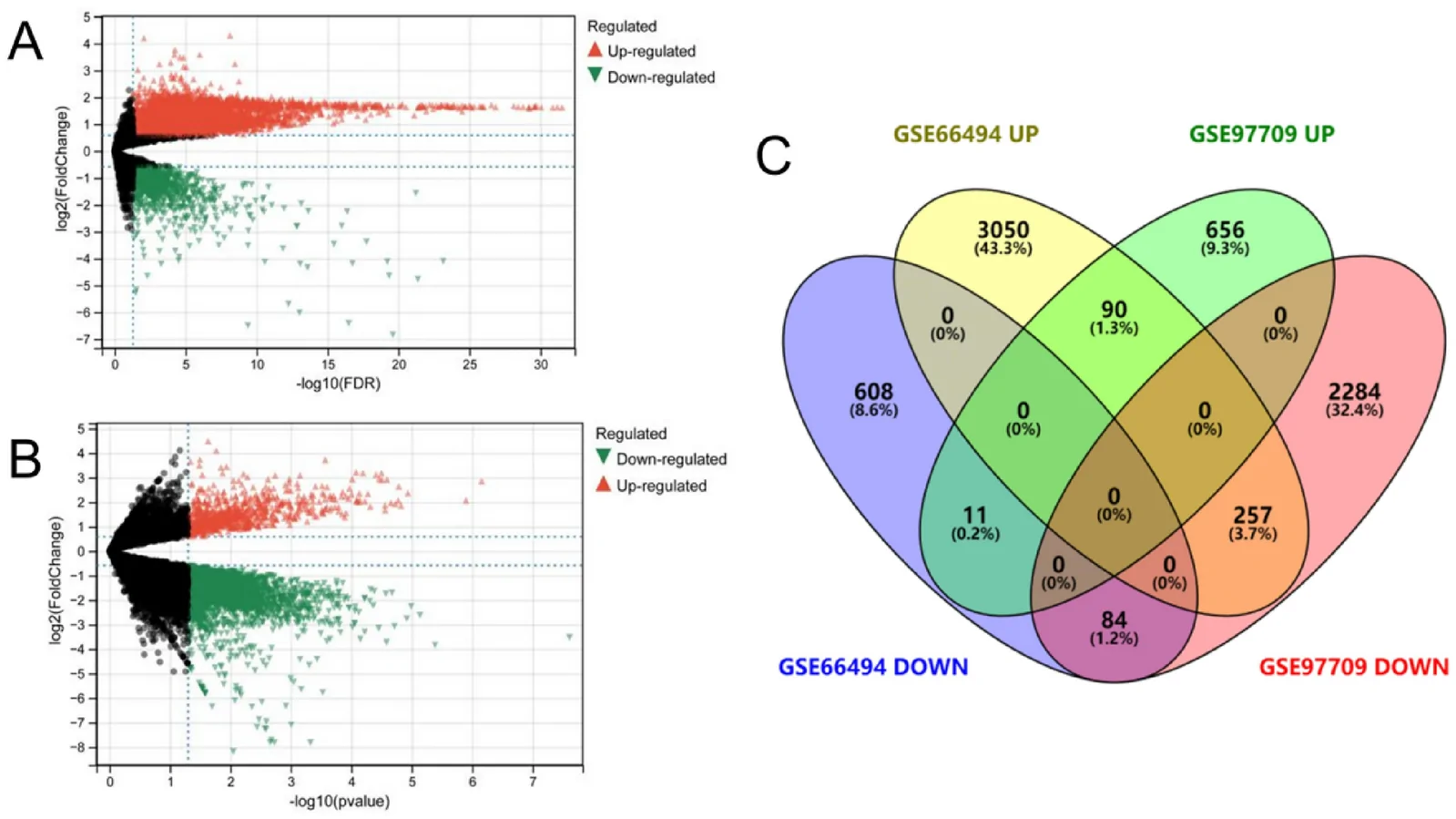
Identification of differentially expressed genes (DEGs). **A, B** Volcano plots of differentially expressed genes (DEGs) for the GSE66494 and GSE97709 datasets, respectively. The *x*-axis indicates log_2_ (fold change, FC), and the *y*-axis represents –log_10_ (false discovery rate, FDR). Red nodes denote significantly upregulated genes (log_2_ Fold Change (FC) ≥ 1.5, FDR < 0.05), green nodes denote significantly downregulated genes (log_2_ Fold Change (FC) ≥ 1.5, FDR < 0.05). **C** Venn diagram illustrating the intersection of DEGs between the GSE66494 and GSE97709 datasets, identifying a total of 442 common DEGs shared by both cohorts

Initial screening of the GSE66494 training set (6 CKD biopsies vs. 10 controls) revealed 4100 DEGs, dominated by 3397 upregulated transcripts (Fig. 1A). Independent assessment of the GSE97709 dataset (8 CKD vs. 12 controls) confirmed widespread transcriptional perturbation, isolating 3382 DEGs (Fig. 1B). By cross-referencing these datasets, we defined a core molecular signature consisting of 442 common DEGs (90 upregulated and 84 downregulated) shared across different platforms and sample types (Fig. 1C). These conserved genes were prioritized for subsequent functional decoding.

### Functional annotation highlights immune dysregulation and TNF signaling

**Fig. 2:**
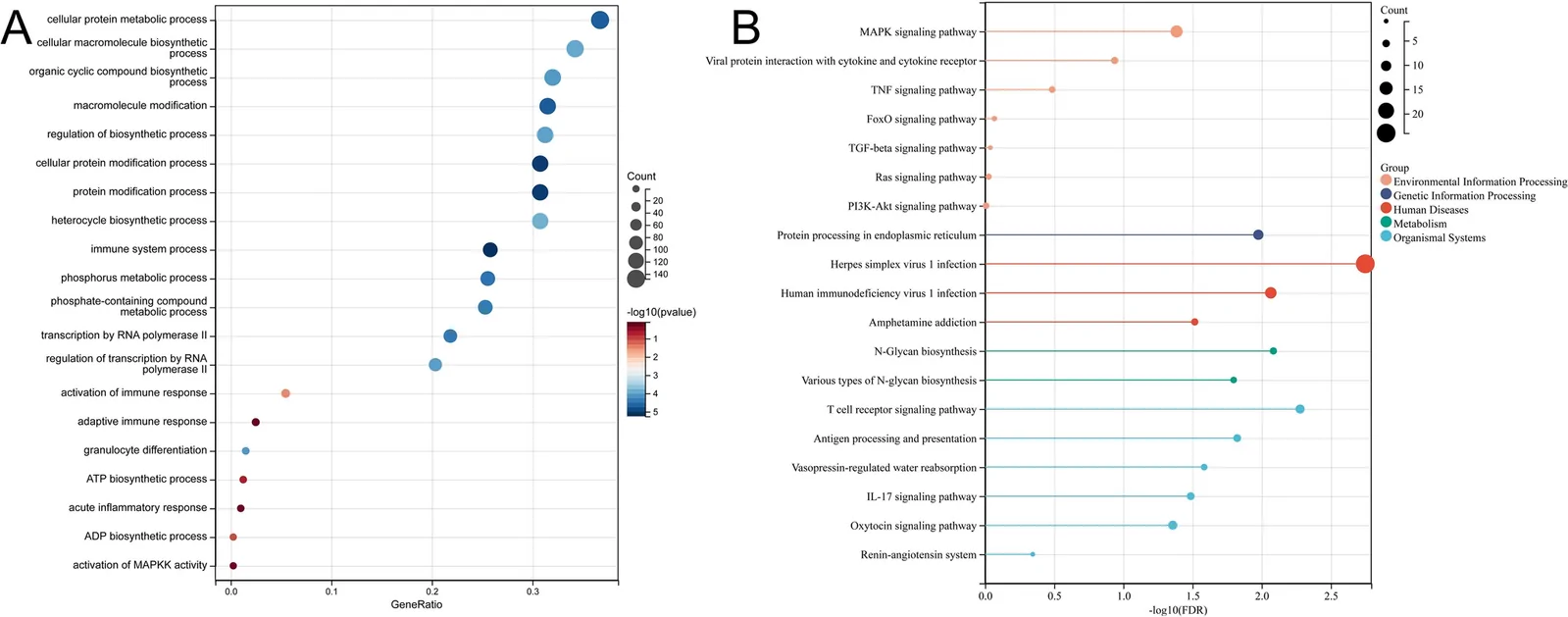
Functional enrichment analyses of the 442 shared DEGs. **A** Bubble plot of Gene Ontology (GO) biological process enrichment. The *Y*-axis lists the biological processes, the *X*-axis represents the gene ratio, and the size of the dots indicates the number of genes enriched in each term. The color gradient (red to blue) represents statistical significance (−log10 *P*-value). **B** Bubble plot of Kyoto Encyclopedia of Genes and Genomes (KEGG) pathway enrichment analysis, highlighting key signaling pathways, such as the TNF signaling pathway and the renin–angiotensin system associated with CKD

Gene ontology (GO) enrichment analysis pointed to profound immune dysregulation in CKD, marked by biological processes driving adaptive immune activation and granulocyte differentiation (Fig. 2A). When mapped against KEGG pathways, these shared DEGs clustered heavily within critical inflammatory signaling cascades. The “TNF signaling pathway” and “FoxO signaling pathway” were among the top hits, alongside the renin–angiotensin system (RAS) (Fig. 2B). These bioinformatic data suggest that immune inflammatory networks act as central engines propelling CKD progression.

### Prioritization of TNFSF14 and CD40 as hub genes

**Fig. 3:**
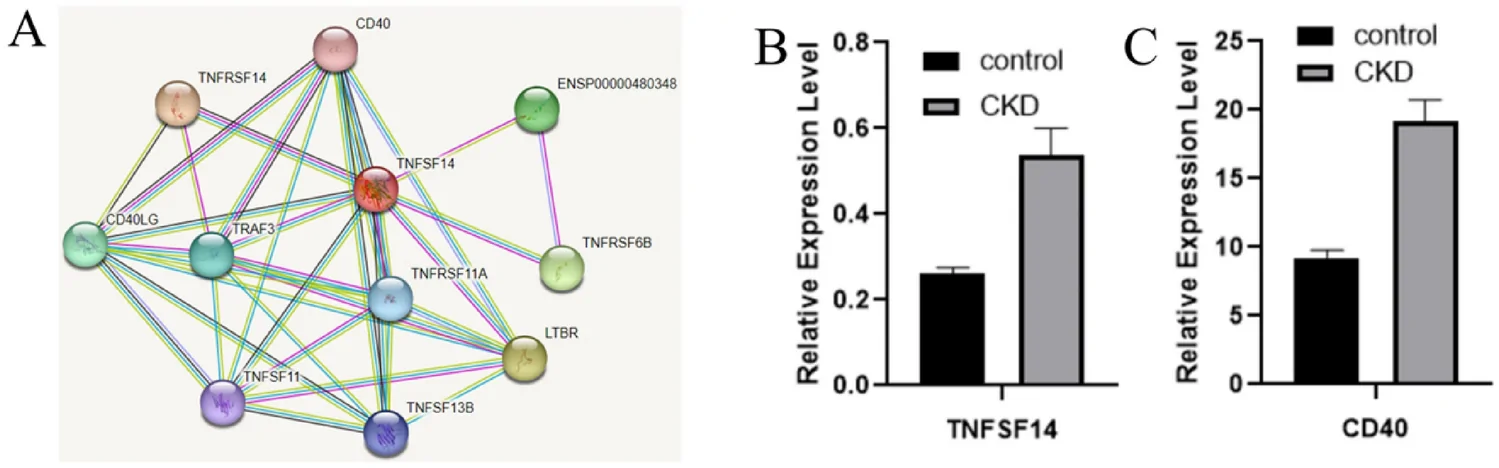
Identification of TNFSF14 and CD40 as key hub genes. **A** Protein–protein interaction (PPI) network constructed using the STRING database, predicting a functional interaction between TNFSF14 and CD40 among the enriched gene set. **B** Bar graph showing the relative expression levels of TNFSF14 in the GSE97709 dataset compared between the control and CKD groups. **C** Bar graph showing the relative expression levels of CD40 in the GSE97709 dataset compared between the control and CKD groups. Data are presented as mean expression values with standard error

Prompted by the significant enrichment of the TNF signaling pathway, we focused on identifying hub genes within this cascade. TNFSF14 showed consistent, high-magnitude dysregulation across both discovery datasets. Furthermore, protein–protein interaction (PPI) network analysis predicted a direct functional synergy between TNFSF14 and CD40 (Fig. 3A). Validation in the GSE97709 dataset confirmed that mRNA levels for both targets were elevated more than twofold compared to controls (*P* < 0.01; Fig. 3B, C). Consequently, the TNFSF14/CD40 axis was selected for translational validation in our clinical cohort.

### Systemic and urinary elevation reflects corrected disease severity

**Fig. 4:**
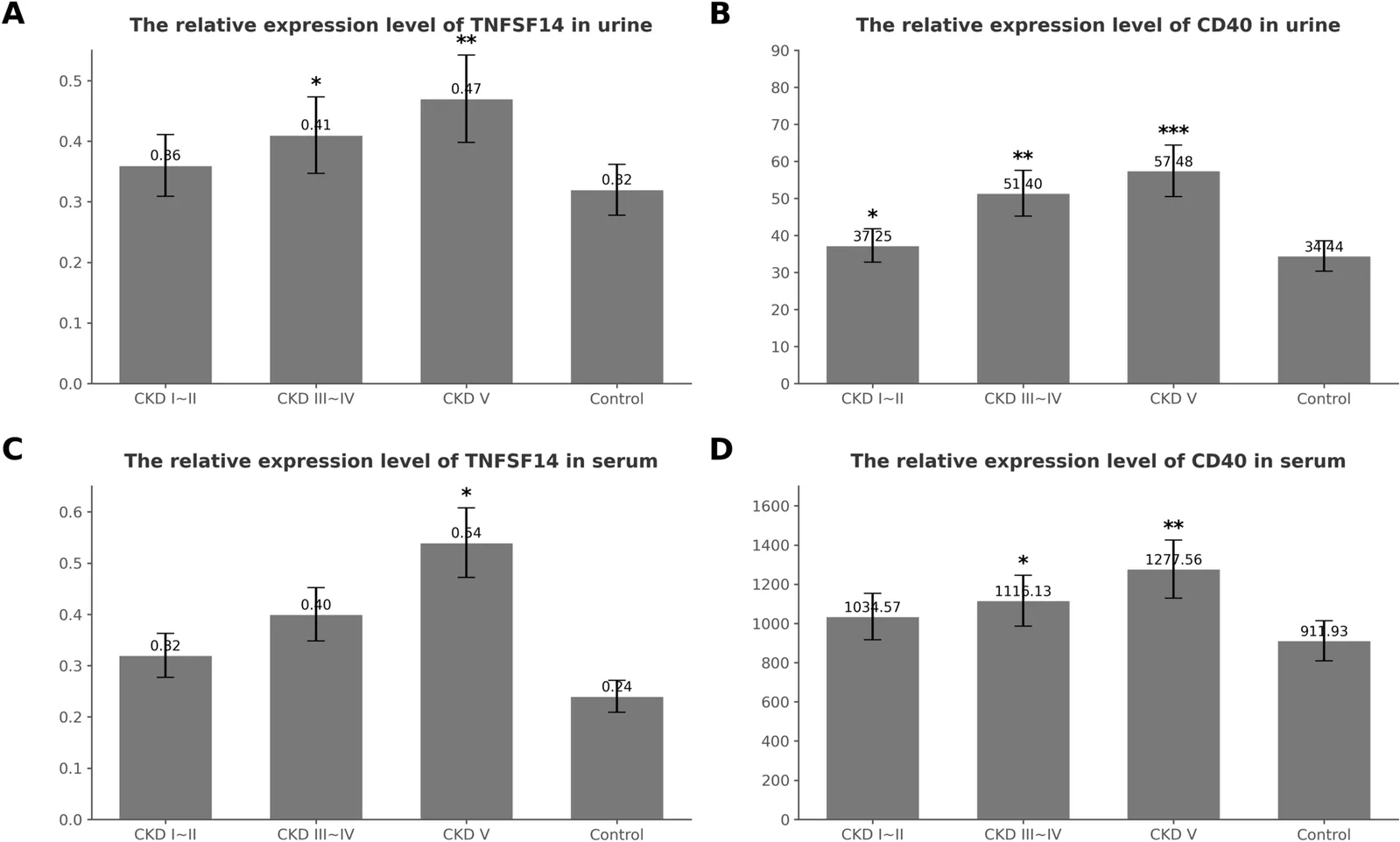
Stage-dependent elevation of TNFSF14 and CD40 in serum and urine across CKD stages. Bar charts illustrate the mean concentrations of urinary (**A, B**) and serum (**C, D**) biomarkers across CKD stages (I–II, III–IV, and V) compared to healthy controls. A clear stepwise increase in both biomarkers tracks with disease progression. Error bars represent standard deviations (SD). *P*-values were determined via ANCOVA, strictly adjusting for clinical confounders including age, gender, and BMI. Asterisks denote statistical significance based on adjusted *P*-values (*P < 0.05, **P < 0.01, ***P < 0.001 versus the control group)

Quantification of protein levels in serum and urine (140 CKD patients vs. 60 healthy controls) via ELISA revealed a marked elevation of both TNFSF14 and CD40 in the patient cohort (*P* < 0.05; Fig. 4). There were no significant differences in age, gender, or BMI distribution between the CKD and control groups (*P* > 0.05), ensuring baseline comparability between the two groups.

Intriguingly, the elevation of both biomarkers followed a clear stage-dependent pattern, with levels increasing progressively alongside advancing CKD stage. After adjusting for age, gender, and BMI via ANCOVA, urinary TNFSF14 and CD40 levels showed a stepwise increase from CKD Stage I–II to Stage V, with statistically significant differences between each CKD stage and the control group (Adjusted *P* < 0.001; Fig. 4A, B). Serum levels of both biomarkers mirrored this stage-dependent increasing trend, with the highest levels observed in patients with end-stage CKD (Stage V) (Fig. 4C, D). These kinetic expression profiles indicate that TNFSF14 and CD40 are not static binary markers of kidney injury, but rather dynamic indicators that reflect the severity of CKD progression.

### Multivariable associations with clinical indicators of renal function

To ensure statistical rigor, associations between these molecular markers and standard clinical indices were evaluated using partial correlation analysis. This model explicitly controlled for major systemic confounders: age, gender, diabetes mellitus, and hypertension (Table 1).

**Table 1:** Partial correlation analysis linking serum and urinary TNFSF14/CD40 concentrations with key clinical and biochemical parameters in CKD patients

Clinical indicators	CD40 in serum	CD40 in urine	TNFSF14 in serum	TNFSF14 in urine
24-h urine protein (g)	−0.069	−0.075	−0.043	−0.120
Hemoglobin (g/L)	−0.118	−0.316**	−0.179*	−0.163
Blood urea nitrogen (mmol/L)	0.139	0.301**	0.162	0.298**
Blood creatinine (μmol/L)	0.150	0.345**	0.287**	0.244**
eGFR	−0.132	−0.438**	−0.158	−0.168*
Blood cystatin C (mg/L)	0.161	0.411**	0.196*	0.269**
Serum β2-microglobulin (mg/L)	0.186*	0.457**	0.233**	0.251**
Parathyroid hormone (ng/L)	0.153	0.306**	0.235**	0.330**
CRP (mg/L)	0.101	0.301**	0.048	0.038

Values represent partial correlation coefficients adjusted for age, gender, diabetes mellitus, and hypertension. Statistical significance is defined after Bonferroni correction for multiple testing

*Adjusted *P* < 0.05, **Adjusted *P* < 0.01

Regarding serum associations, serum TNFSF14 and CD40 tracked positively with retention markers (creatinine, cystatin C, β2-MG) and parathyroid hormone (PTH), while demonstrating an inverse relationship with hemoglobin. Crucially, the urinary output of both TNFSF14 and CD40 displayed strong, positive, and independent correlations with key injury markers (BUN, cystatin C, β2-MG) and highly significant negative correlations with eGFR. The persistence of these moderate-to-strong associations post-Bonferroni adjustment reinforces their clinical relevance as specific, rather than spurious, indicators of functional renal decline.

### Multivariable risk assessment and diagnostic superiority

**Fig. 5:**
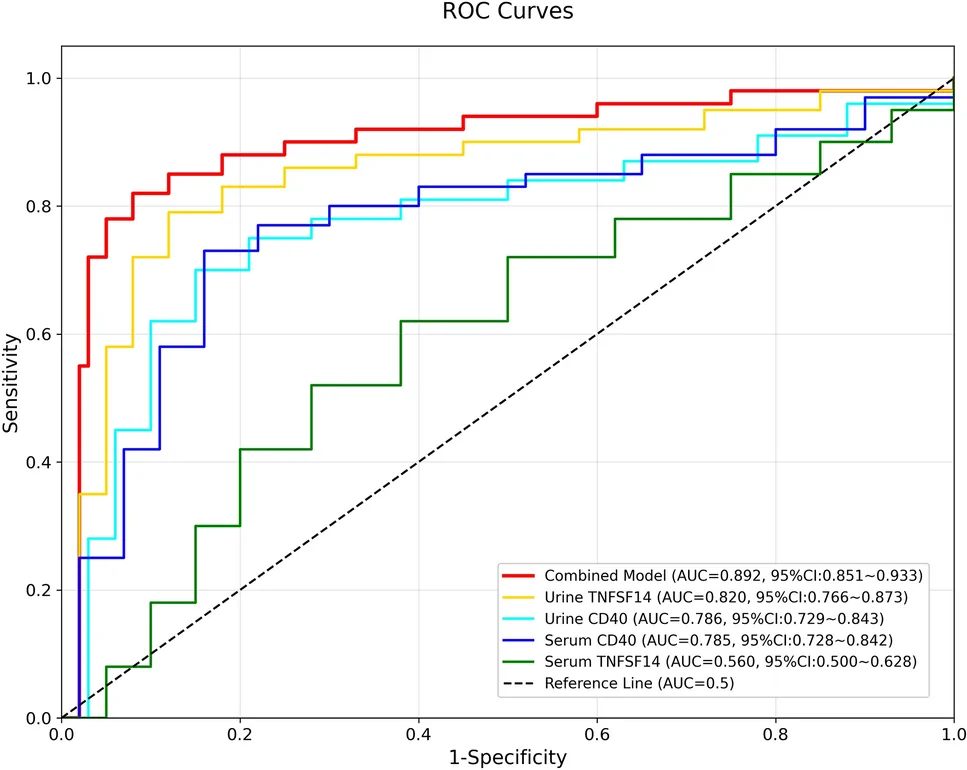
Diagnostic performance via ROC analysis. Comparison of ROC curves for individual markers (serum CD40, urinary CD40, serum TNFSF14, and urinary TNFSF14) and a Combined Multivariable Model (red line). The inclusion of clinical covariates enhances the Area Under the Curve (AUC) to 0.892 (95% CI: 0.851–0.933), demonstrating superior diagnostic robustness over single indicators

ROC curve analysis was performed to evaluate the diagnostic performance of serum and urinary TNFSF14 and CD40 for CKD (Fig. 5). Urinary biomarkers showed superior diagnostic accuracy compared to their serum counterparts: urinary TNFSF14 achieved an AUC of 0.820 (95% CI: 0.766–0.873), and urinary CD40 achieved an AUC of 0.786 (95% CI: 0.729–0.843). Serum CD40 showed moderate diagnostic performance with an AUC of 0.785 (95% CI: 0.728–0.842), while serum TNFSF14 showed poor diagnostic performance with an AUC of 0.560 (95% CI: 0.500–0.628). Notably, a combined diagnostic model integrating urinary TNFSF14, urinary CD40, and clinical covariates (age, gender, diabetes, hypertension) achieved a significantly improved AUC of 0.892 (95% CI: 0.851–0.933), outperforming any single biomarker alone.

**Fig. 6:**
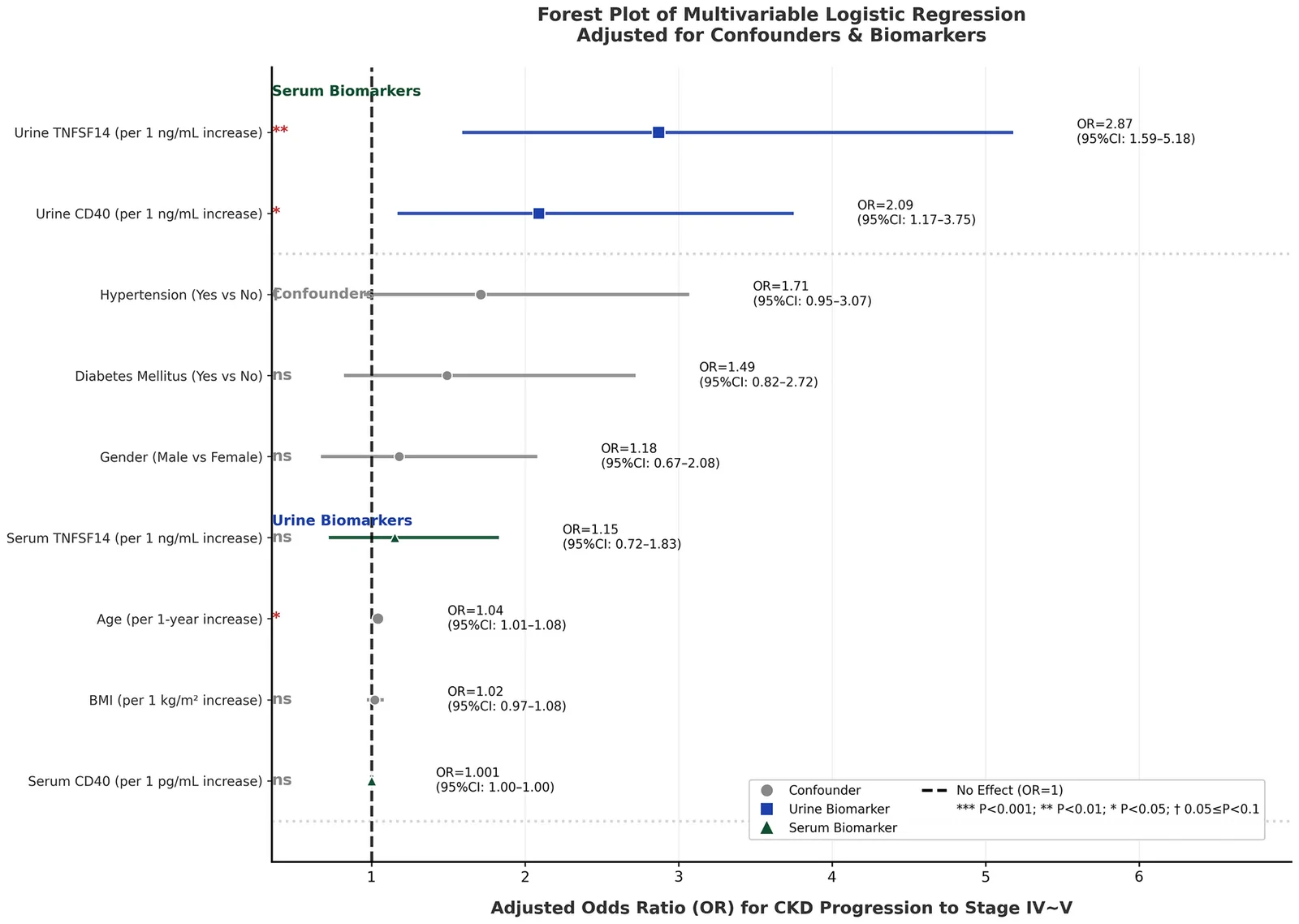
Immunofluorescence staining of TNFSF14 and CD40 in renal tissues from patients with different pathological types of CKD. **A** Representative immunofluorescence image of TNFSF14 staining (red) in renal tissues from healthy controls and patients with Membranous Nephropathy (MN), IgA Nephropathy (IgAN), Diabetic Nephropathy (DN), and Focal Segmental Glomerulosclerosis (FSGS). Nuclei were counterstained with DAPI (blue). Images are shown at 200× (upper row) and 400× (lower row) magnification. **B** Representative immunofluorescence image of CD40 staining (red) in control and CKD renal tissues (MN, IgAN, DN, FSGS). Positive expression was predominantly observed in the renal tubular region

Multivariable logistic regression analysis was further performed to determine the independent predictive value of TNFSF14 and CD40 for advanced CKD (Stages IV–V), with results visualized in a forest plot (Fig. 6). The regression model showed excellent goodness-of-fit (Hosmer–Lemeshow test *P* = 0.48) and robust explanatory power (McFadden pseudo *R*^2^ = 0.27). After adjusting for demographic and metabolic confounders, age was identified as a significant independent risk factor for advanced CKD (Adjusted OR = 1.04, 95% CI: 1.01–1.08, *P* = 0.021), while gender, BMI, diabetes, and hypertension did not reach statistical significance.

Critically, the regression analysis revealed a clear divergence between urinary and serum biomarkers: urinary TNFSF14 was the strongest independent predictor of advanced CKD, with each 1-unit increase in urinary TNFSF14 associated with a 187% increased risk of advanced CKD (Adjusted OR = 2.87, 95% CI: 1.59–5.18, *P* < 0.001). Urinary CD40 also remained a significant independent predictor of advanced CKD (Adjusted OR = 2.09, 95% CI: 1.17–3.75, *P* = 0.013). In stark contrast, neither serum TNFSF14 nor serum CD40 showed independent predictive value for advanced CKD after adjustment for urinary biomarkers and clinical covariates (*P* > 0.05 for both). These findings confirm that urinary, rather than circulating, TNFSF14 and CD40 are robust independent predictors of CKD progression.

### Tubulointerstitial localization of target proteins

**Fig. 7:**
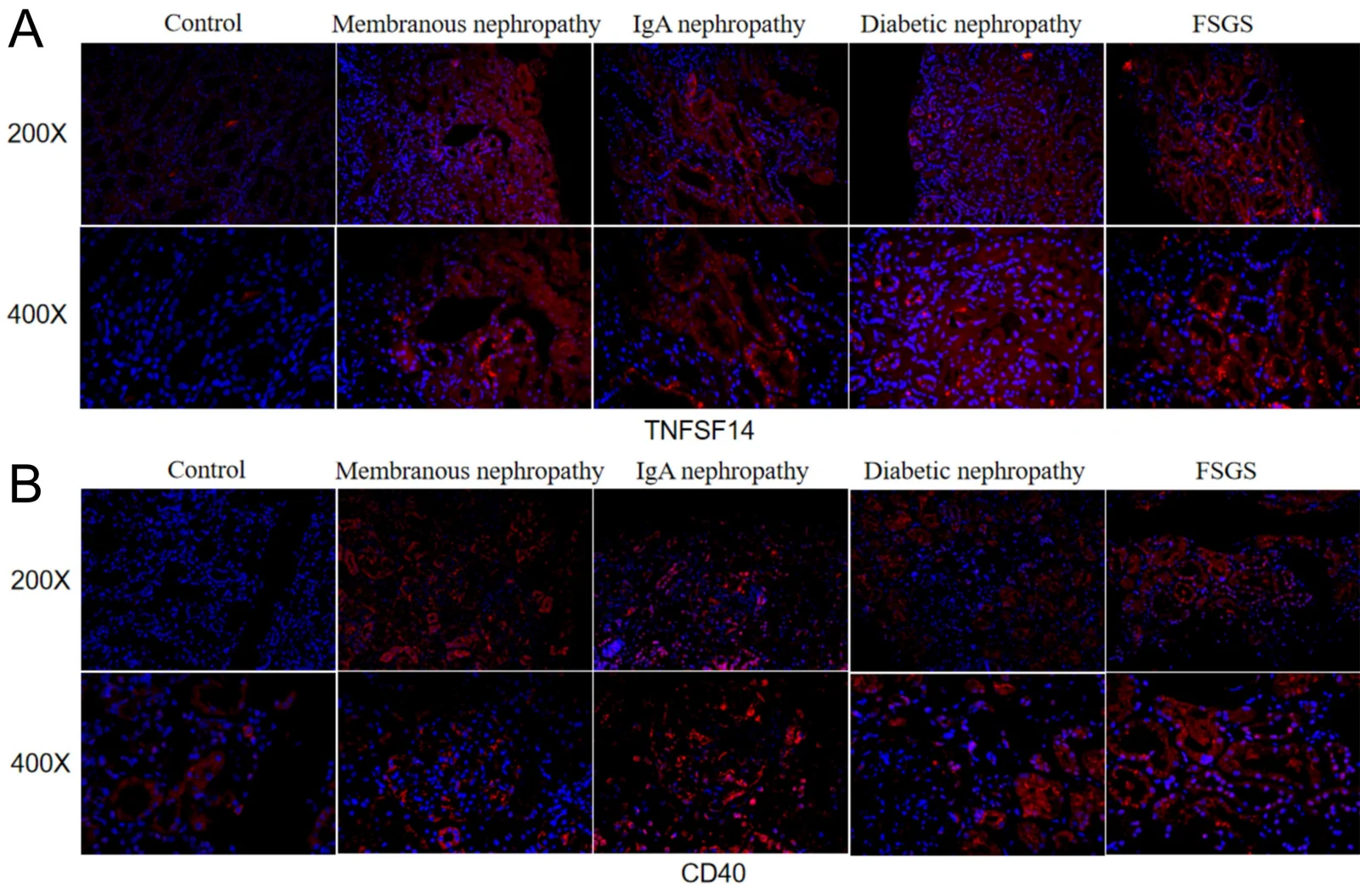
Quantitative analysis of immunofluorescence intensity for TNFSF14 and CD40 in renal tissues. **A** Quantification of the mean fluorescence intensity of TNFSF14 in the control and various CKD pathological groups. **B** Quantification of the mean fluorescence intensity of CD40. Data are expressed as mean ± SD

**Fig. 8:**
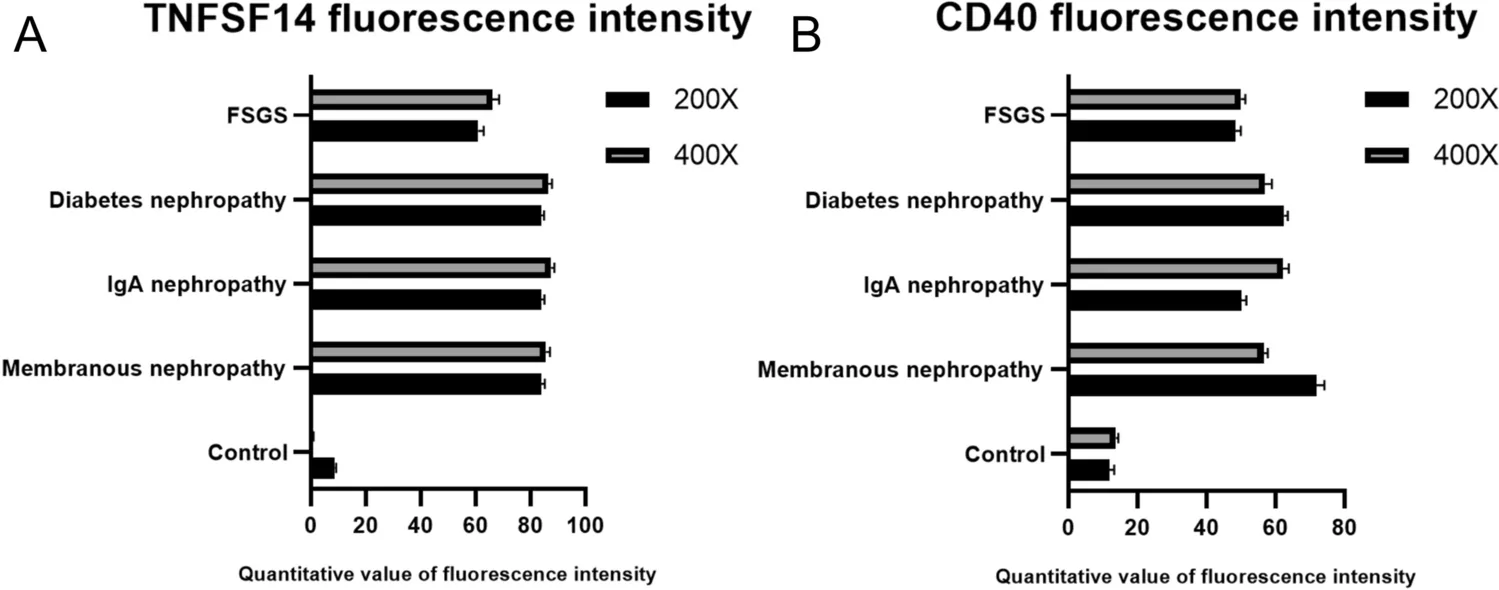
Multivariable Logistic Regression Forest Plot of independent risk factors. The plot illustrates Adjusted Odds Ratios (OR) and 95% Confidence Intervals (CI) for urinary and serum biomarkers alongside clinical covariates. The model demonstrated excellent fit (Hosmer–Lemeshow test *P* = 0.48). Urinary TNFSF14 (OR = 2.87, *P* < 0.001) and CD40 (OR = 2.09, *P* = 0.013) are identified as robust independent predictors, significantly outperforming their serum counterparts (*P* > 0.05). Age was included as a significant clinical confounder (*P* = 0.021). The vertical dashed line represents the null hypothesis (OR = 1)

To define the spatial context, immunofluorescence staining was performed on biopsies covering major CKD etiologies (DN, IgAN, MN, FSGS). In contrast to the negligible signal in normal tissues, prominent fluorescence for both TNFSF14 and CD40 was observed across all diseased samples (Fig. 7). Microscopically, this upregulation was specifically localized to the renal tubular epithelial cells and the tubulointerstitial compartment. Quantitative analysis confirmed significantly higher fluorescence intensity in all CKD groups (Fig. 8), identifying tubulointerstitial overexpression of the TNFSF14/CD40 axis as a universal pathological feature, independent of the primary glomerular injury.

## Discussion

The clinical trajectory of chronic kidney disease is defined by a progressive decline toward tubulointerstitial fibrosis and irreversible nephron loss. With the global prevalence of CKD accelerating, there is an operational imperative to transition from traditional late-stage diagnostics to early molecular monitoring models [1, 2, 9]. While renal biopsy remains the pathological gold standard, its invasive nature precludes frequent longitudinal repetition [4, 11]. By bridging rigorous transcriptomic bioinformatics with independent clinical validation, this study identifies TNFSF14 and CD40 as biologically significant indicators of tubulointerstitial stress. Crucially, these molecules are universally upregulated in damaged renal tubules and accurately detectable in urine, thereby providing a non-invasive analytical diagnostic modality into the active inflammatory status of the kidney [11, 16].

The transcriptomic screening, fortified by strict multiple testing corrections, positioned the TNF signaling pathway as a dominant orchestrator of the transcriptional landscape in CKD. This finding aligns robustly with the prevailing paradigm of inflammation-driven renal fibrosis [6, 12]. Within this signaling cascade, TNFSF14 and CD40 emerged as pivotal hub genes. Historically categorized primarily as a T cell co-stimulator, TNFSF14 is increasingly recognized as a central mediator in tissue remodeling [13]. Experimental evidence suggests that TNFSF14 impairs endothelial progenitor cell function, thereby restricting renal neovascularization and exacerbating the hypoxic microenvironment that accelerates CKD progression [17, 18]. Such vascular–immune crosstalk implies that elevated TNFSF14 levels reflect a dual pathology involving both active inflammation and maladaptive vascular repair.

Parallel to these findings, the detection of CD40 overexpression specifically in renal tubules challenges historical views of the molecule as an exclusive B cell marker. This observation is consistent with recent high-dimensional spatial profiling of human biopsies, which identifies CD40+ tubular cells and macrophages as integral components of the fibrotic niche [5, 10, 19]. Mechanistically, the ligation of CD40 on tubular epithelial cells triggers the profound release of pro-inflammatory chemokines, establishing a feed-forward loop that perpetuates leukocyte recruitment into the interstitium [14, 15]. The immunohistochemical data presented herein corroborate this mechanism, revealing intense CD40 staining specifically within the tubular compartment across distinct etiologies, including DN, IgAN, and MN. This indicates that tubular CD40 expression represents a universal stress response to renal injury, independent of the initiating primary disease trigger [16].

Beyond leukocyte recruitment, the specific upregulation of TNFSF14 and CD40 in tubular epithelial cells suggests a direct contribution to fibrogenic remodeling [20, 21]. Ligation of CD40 on renal tubular cells acts as a potent activator of the NF-κB signaling pathway. This activation sustains a chronic inflammatory milieu and can transcriptionally repress E-cadherin while upregulating mesenchymal markers [21]. Consequently, tubular cells may lose their polarity, acquiring a profibrotic phenotype that actively deposits extracellular matrix. Therefore, the high accumulation of these proteins in the tubulointerstitium is likely an active driver that accelerates the transformation of functional tubules into scar tissue [16, 21].

A pivotal translational aspect of this work is the validation of these targets as non-invasive urinary biomarkers. The robust partial correlation observed between urinary TNFSF14/CD40 and β2-microglobulin (β2-MG)—a highly specific index of tubular dysfunction—supports the hypothesis that these proteins are shed directly from damaged tubular epithelium rather than being passively filtered from systemic circulation [11, 22]. This distinction is clinically vital as tubulointerstitial injury often predicts long-term renal survival more accurately than initial glomerular markers [16]. Our multivariable logistic regression analysis unequivocally reinforced this biological paradigm. By demonstrating that urinary TNFSF14 (OR = 2.87) and urinary CD40 (OR = 2.09) independently predict advanced CKD, our model confirms their stability against classical confounding factors, such as diabetes, hypertension, and BMI [7, 8]. Furthermore, the complete loss of independent predictive value for serum TNFSF14 and CD40 in the adjusted model serves as a critical internal control. It strongly suggests that while systemic inflammation (reflected in serum) accompanies CKD, the localized, tubulointerstitial pool (reflected in urine) is the true, independent indicator of structural renal decline [23]. Given their favorable diagnostic performance (Combined AUC = 0.892), the quantification of these urinary markers could be instrumental in monitoring residual inflammatory risk in patients who present with stable serum creatinine but harbor ongoing, occult tubulointerstitial damage [4, 24, 25].

Extending the analysis to systemic complications, significant multivariable associations were noted between these biomarkers and extra-renal manifestations. The positive correlation between serum TNFSF14 and Parathyroid Hormone (PTH) provides clinical support for the interaction between inflammatory cytokines and the RANKL/OPG system within the kidney-bone axis [26]. Furthermore, the inverse relationship with hemoglobin levels implies a potential connection between the TNFSF14/CD40 axis and inflammatory renal anemia as inflammatory cytokines are known to suppress erythropoietin and induce hepcidin [27].

While this study provides compelling evidence for the diagnostic utility of TNFSF14 and CD40, several limitations necessitate cautious interpretation. First, the cross-sectional design inherent to the clinical validation cohort establishes strong associations but precludes definitive inferences regarding causality. It remains to be determined in longitudinal frameworks whether the elevation of these biomarkers strictly precedes the decline in eGFR [7, 8]. Second, the GEO datasets utilized differ significantly in analytical platforms (microarray vs. RNA-seq). While stringent batch effect corrections via ComBat were applied, inherent technical variance remains a factor [28]. Third, the cohort was restricted to adult patients from a single center; thus, the applicability of these findings to pediatric CKD populations or across diverse ethnicities requires independent multicenter validation [29, 30]. Finally, although immunofluorescence confirmed spatial tissue localization, functional in vitro or in vivo mechanistic experiments—such as siRNA knockdown or CRISPR/Cas9 ablation of the TNFSF14/CD40 axis in human renal tubular cells—were not performed. Future mechanistic assays are essential to fully dissect the intracellular signaling events downstream of this axis to conclusively evaluate its therapeutic potential [3, 31–33].

## Conclusion

By integrating rigorous transcriptomic bioinformatics with multivariable clinical validation, this study identifies TNFSF14 and CD40 as biologically relevant, stage-dependent biomarkers for Chronic Kidney Disease. The data underscore the centrality of the TNF signaling axis in driving renal pathology. Specifically, the universal upregulation of these proteins within the tubular compartment, coupled with their progressive elevation in urine, provides a precise reflection of tubulointerstitial disease severity. Multivariable evaluation establishes urinary TNFSF14 and CD40 as robust, independent predictors of advanced CKD, significantly outperforming their systemic counterparts regardless of patient age or metabolic comorbidities. These results position urinary TNFSF14 and CD40 as actionable candidates for non-invasive diagnostic monitoring, potentially guiding future immunomodulatory interventions aimed at curbing renal inflammation and delaying the clinical trajectory of kidney failure.

## Supplementary Information

Below is the link to the electronic supplementary material.

Supplementary file1 (XLSX 11 KB)

## Data Availability

The data supporting this study’s findings are available from the corresponding author or Xiameng Gu, upon reasonable request.
